# MICaFVi: A Novel Magnetic Immuno-Capture Flow Virometry Nano-Based Diagnostic Tool for Detection of Coronaviruses

**DOI:** 10.3390/bios13050553

**Published:** 2023-05-18

**Authors:** Nosaibah Samman, Kheireddine El-Boubbou, Khawlah Al-Muhalhil, Rizwan Ali, Ahmed Alaskar, Naif Khalaf Alharbi, Atef Nehdi

**Affiliations:** 1 Medical Research Core Facility and Platforms (MRCFP), King Abdullah International Medical Research Center (KAIMRC) & King Saud bin Abdulaziz University for Health Sciences (KSAU-HS), King Abdulaziz Medical City (KAMC), Ministry of National Guard Health Affairs (MNGHA), Riyadh 11426, Saudi Arabia; 2 King Abdullah International Medical Research Center (KAIMRC) & King Saud bin Abdulaziz University for Health Sciences (KSAU-HS), King Abdulaziz Medical City (KAMC), Ministry of National Guard Health Affairs (MNGHA), Riyadh 11426, Saudi Arabia; 3 Nanomaterials for Bioimaging Group (nanoBIG), Facultad de Ciencias, Departamento de Física de Materiales, Universidad Autónoma de Madrid (UAM), 28049 Madrid, Spain; 4 Department of Chemistry, College of Science, University of Bahrain, Sakhir 32038, Bahrain; 5 Department of Oncology, King Abdulaziz Medical City, College of Medicine, King Saud bin Abdulaziz University for Health Sciences (KSAU-HS), Ministry of National Guard Health Affairs (MNGHA), Riyadh 11426, Saudi Arabia; 6 Department of Life Sciences, Faculty of Sciences of Gabes, University of Gabes, Gabes 6029, Tunisia

**Keywords:** nano-based sensor, immuno-capture, flow-cytometry, detection, coronavirus, MERS-CoV, SARS-CoV-2

## Abstract

COVID-19 has resulted in a pandemic that aggravated the world’s healthcare systems, economies, and education, and caused millions of global deaths. Until now, there has been no specific, reliable, and effective treatment to combat the virus and its variants. The current standard tedious PCR-based tests have limitations in terms of sensitivity, specificity, turnaround time, and false negative results. Thus, an alternative, rapid, accurate, and sensitive diagnostic tool that can detect viral particles, without the need for amplification or viral replication, is central to infectious disease surveillance. Here, we report MICaFVi (**M**agnetic **I**mmuno-**C**apture **F**low **V**irometry), a novel precise nano-biosensor diagnostic assay for coronavirus detection which combines the MNP-based immuno-capture of viruses for enrichment followed by flow-virometry analysis, enabling the sensitive detection of viral particles and pseudoviruses. As proof of concept, virus-mimicking spike-protein-coated silica particles (VM-SPs) were captured using anti-spike-antibody-conjugated MNPs (AS-MNPs) followed by detection using flow cytometry. Our results showed that MICaFVi can successfully detect viral MERS-CoV/SARS-CoV-2-mimicking particles as well as MERS-CoV pseudoviral particles (MERSpp) with high specificity and sensitivity, where a limit of detection (LOD) of 3.9 µg/mL (20 pmol/mL) was achieved. The proposed method has great potential for designing practical, specific, and point-of-care testing for rapid and sensitive diagnoses of coronavirus and other infectious diseases.

## 1. Introduction

A novel coronavirus strain, SARS-CoV-2, was first identified in Wuhan, China, in December 2019, resulting in a coronavirus disease (COVID-19) pandemic [1,2], infecting ~750 million people and leading to more than 6.8 million deaths globally, as reported by WHO [3]. The pandemic was considered to be one of the deadliest crises in history, in which the world economy underwent severe negative and long-lived consequences. Due to the absence of specific treatments for COVID-19, governments have primarily focused on preventing the spread of the disease instead of relying on therapeutics or repurposed drugs. This has involved measures such as lockdowns, home quarantine, social-distancing, and self-isolation for up to two weeks after receiving a positive PCR result. Moreover, the development of detection techniques such as immuno-assays and “test-at-home” kits, as well as vaccination campaigns, emerged [4]. With healthcare systems struggling to cope with the pandemic, the significance of alternative, efficient, sensitive, and selective diagnostic tools cannot be overstated. 

Recently, the genome sequences of SARS-CoV-2 and MERS-CoV have been revealed, showing that SARS-CoV-2 is about 82% identical to the original SARS-CoV-2002 and MERS-CoV-2012, with over 90% similarity in sequences that encode essential enzymes and structural proteins [5,6]. The use of techniques, particularly real-time reverse transcription polymerase chain reaction (RT-PCR), has been widely utilized and they have been developed as kits for clinical diagnosis [7]. RT-PCR is a real-time test which quantitatively detects the nucleic acid viral RNA of the viruses present in upper and lower respiratory specimens. Although it is considered the gold standard for diagnosis, PCR techniques (RT-PCR or RT-qPCR) have certain limitations. They suffer from a high rate of false negative results, particularly during the first week of infection before it turns positive [8,9,10]. During this period, patients can be infectious. Moreover, sample extraction and test preparation need a long turnaround time, rendering the sample processing time lengthy. The method takes 3–6 h from sample collection to result generation, and due to the huge number of requested tests, the time required to obtain the results can be up to 2–3 days [11]. Moreover, RT-qPCR-based testing requires trained personnel, sophisticated laboratory infrastructure, biosafety cabinets, and thermocyclers, which are not available in most healthcare settings, especially in underdeveloped and developing countries. The sensitivity and specificity of RT-PCR- or RT-qPCR-based diagnostics are tightly dependent on several factors, including sample quality and type, the timing of sample collection, the type of PCR assay used, the viral load, and the presence of inhibitors or mutations in the viral genome [12,13,14,15]. This explains the relatively high rate of false negative and/or false positive results obtained, and consequently the need for multiple confirmatory testing, especially in mild cases where samples can test negative due to a low viral load [16,17,18,19]. Importantly, RT-qPCR diagnostic kits are expensive and not always available (i.e., during the COVID-19 pandemic many countries suffered from a shortage). In addition, the emergence of virus variants has created difficulties in accurately diagnosing patients. The mutations in these strains have led to higher rates of false negative results and greater variation in the clinical manifestations of the disease [20]. Thus, it is imperative to find alternative approaches to detect SARS-CoV-2 and MERS-CoV viruses in a reliable, efficient, and cost-effective way without jeopardizing specificity and sensitivity. As new variants of the virus continue to emerge, the need for accurate and adaptable diagnostic methods is becoming more urgent. Given the continuous need for fast, sensitive, and cost-effective viral detection techniques, the advantages of using nano-based sensors are particularly promising.

Since the emergence of SARS-CoV-2, numerous researchers and scientific experts have been exploring the potential of nanoparticle (NP)-based approaches for the efficient detection of the virus [21,22,23,24]. In fact, NP-based methodologies have recently been found to be very effective in rapid detection, sensing, improvements, prevention (i.e., masks and personal protective equipment), vaccine development, and accessible automated viral testing [25]. In general, the use of nanomaterials has been reported to decrease detection time while increasing sensitivity, making it possible to design improved detection approaches for coronaviruses [23,26]. The unique NP physicochemical properties, such as the magnetic properties of magnetic nanoparticles (MNPs), plasmonic properties of gold nanoparticles (AuNPs), electrochemical properties of carbon-based nanoparticles, and fluorescence properties of quantum dots, can be tailored according to their specific application requirements, making them a desirable platform for rapid, specific, and low-cost detection [21,22]. Furthermore, surface functionalization with specific ligands, such as antibodies, peptides, proteins, etc., can improve the detection limit and specificity. Spike and nucleocapsid proteins, among others, are the most promising targets for antigen-based diagnostic tests [27,28]. In particular, the spike S protein is the antigen that has been studied the most. This protein attaches to the angiotensin-converting enzyme 2 (ACE2) receptor, which is typically present in epithelial linings, such as the respiratory and gastrointestinal tracts, thereby enabling the virus to enter and infect the host cells [29,30]. Different point-of-care diagnostic tools and detection techniques have been utilized, including electrochemical, optical, electrochemiluminescence, photoluminescence, colorimetric, immuno-based sensing, and magnetic nano-sensing [22,23,31]. In particular, MNPs have shown great potential as detection tools for various virus types. The main advantages of using MNPs for such purposes are as follows: small sizes, facile functionalization, excellent biocompatibilities and stabilities, low toxicities, and superior magnetic responsiveness [32]. Most magnetic biosensors fall into major categories, namely, magnetoresistance (MR), magnetic particle spectroscopy (MPS), and nuclear magnetic resonance (NMR) platforms [33]. These materials can be functionalized using specific biotags to capture viruses and target analytes selectively and specifically from complex biological samples, and their magnetic properties can be utilized for efficient separation and purification. In this regard, both surface-(functionalized on surface) and volume-based (dispersed in liquid) magnetic sensing have been exploited to aid in the detection of viruses and pathogens [33]. For instance, the detection of SARS-CoV-2 mimicking using anti-SARS-CoV-2-spike-monoclonal-antibody-functionalized MNPs (80 nm Dextran-coated Bionized NanoFerrite (BNF) particles) was achieved via the measurement of their magnetic responses under an AC magnetic field [34]. The proposed approach allowed for rapid detection (~1 h) with a limit of detection of 0.084 nM. Later on, a five-minute MPS-based bioassay using anti-SARS-CoV-2-spike-polyclonal-antibody-functionalized MNPs (30 nm IPG30 NPs) for the ultrafast detection of the SARS-CoV-2 spike protein at a higher temperature (37 °C) with a detection limit ~5 nM was reported [35]. In another elegant piece of work, Seo et al. illustrated a field-effect transistor (FET)-based biosensor to detect SARS-CoV-2 in clinical samples [36]. The FET biosensor produced by coating graphene sheets of FET with a specific antibody against the SARS-CoV-2 spike protein detected the virus antigen protein with high sensitivity (LOD = 100 fg/mL in biological samples), using only small amounts of analytes. In addition to the above platforms, other platforms that utilize MNPs as auxiliary tools for detecting viruses have also been reported. The combination of MNPs with various analytical techniques (i.e., PCR, ELISA, mass spectrometry, and flow cytometry) has led to an advancement in viral detection techniques. One example is depicted by Chou et al. using viral-antibody-functionalized MNPs as efficient magnetic separation probes for rapid and sensitive virus detection using mass spectroscopy [37]. Pietschmann et al. also demonstrated a portable MInD (magnetic immuno-detection) surface-based immuno-assay approach for SARS-CoV-2 S-protein peptide specific antibody detection in spiked human serum [38]. Furthermore, MNPs have been frequently coupled with several other non-magnetic materials such as Au, silver (Ag), silica, fluorescent probes, and quantum dots in different bioassay platforms. For instance, Zhao et al. elegantly reported an ultrasensitive supersandwich-type Au@Fe_3_O_4_/graphene oxide host–guest complexed sensor for SARS-CoV-2 detection in infected COVID-19 patients using a portable electrochemical smartphone [39]. Individual Au and Ag NP-based testing systems were also investigated for SARS-CoV-2 and other forms of viral detection [40]. One commonly used point-of-care (POC) diagnostic tool in this regard is the lateral flow assay (LFA). Examples of SARS-CoV-2 LFA devices utilizing AuNPs for detecting SARS-CoV-2 spike or nucleocapsid proteins have been developed with detection limits ranging from 0.65 ng/mL to 5 μg/mL [41]. However, LFAs are limited in their specificity, sensitivity, or possibility for quantitative measurements. Moreover, the performance and efficacy of the most promising rapid antigen-based tests for actual applications are still questionable. Thus, there is an urgent need for novel alternative methodologies that are rapid, practical, accurate, sensitive, and easy to use for virus diagnostics.

Herein, we report MICaFVi (**M**agnetic **I**mmuno-**C**apture **F**low **V**irometry), a novel magnetic nano-based diagnostic tool that combines MNP-based immuno-capture of viruses for enrichment followed by flow-virometry analysis for detection. We sought to develop a highly sensitive, accurate, and practical detection technique that does not require tedious nucleic acid extraction or amplification and could be integrated into a rapid, quantitative, and selective automated workflow. MICaFVi incorporates a 30 min to a few hours incubation step followed by a short readout, achieving fast and sensitive detection. For safety reasons, MICaFVi was developed and optimized using virus-mimicking silica-based particles (VM-SPs). Overall, our MICaFVi technique enabled the accurate, quantitative, specific, and sensitive detection of MERS-CoV/SARS-CoV-2-mimicking particles, as well as MERS-CoV pseudoviral particles (MERSpp). The limit of detection (LOD) was found to be equal to 3.9 µg/mL (20 pmol/mL). This quick and accurate diagnosis tool will greatly advance surveillance and control strategies for viral diseases, especially for future pandemics.

## 2. Materials and Methods

Unless otherwise indicated, all chemicals and solvents were obtained from commercial suppliers and used as supplied without further purification. Cetyltrimethyl ammonium bromide (CTAB), sodium fluoride (NaF), tetraethyl orthosilicate (TEOS), N-(trimethoxysilylpropyl)-ethylenediaminetriacetic acid carboxy-triethoxysilane (TEDTA-COOH), 1-ethyl-3-(3-dimethylaminopropyl) carbodiimide (EDC), N-hydroxysulfosuccinimide (NHS-sulfo), and paraformaldehyde (PFA) were all purchased from UFC Biotechnology (Riyadh, Saudi Arabia) and Sigma-Aldrich (MO, USA.). Superparamagnetic iron oxide MNPs were purchased from BOC Sciences (NY, USA), CMB-500N, size ~500 nm. The following antibodies and recombinant proteins were purchased from Sino Biological (Beijing, China): anti-MERS-CoV spike protein S1 mouse monoclonal antibody, FITC-labeled (cat # 40069-MM23); anti-MERS-CoV spike protein S1, Rabbit polyclonal antibody (cat # 40069-T52); and Recombinant MERS-CoV spike protein (cat # 40069-V08H). Donkey anti-Rabbit APC-labeled secondary antibody was purchased from Thermo-Fisher, USA (Cat # A-31573). MERS-CoV pseudoviral particles (MERSpps) were generated and quantified as relative light units per ml (RLU/mL) following established protocols [42]. These pseudovirus particles are equivalent to the HIV lentiviral core, displaying the MERS-CoV spike protein on their surface. The MERSpp genome carries a luciferase gene that will be utilized as an infection reporter. Vesicular Stomatitis Virus-Glycoprotein lentiviral particles (VSV-Gpp) were produced in the lab in HEK293T cells using the Lenti-vpack Lentiviral Packaging kit (OriGene). VSV-G lentivirus particles display the envelope glycoprotein (VSV-G) and contain a heterologous lentiviral (HIV) core. VSV-Gpp expresses the GFP optical reporter gene upon infection. These pseudoviral particles (MERSpp and VSV-Gpp) are replication-incompetent and capable of a single round of infection.

### 2.1. Transmission Electron Microscopy (TEM)

Samples were prepared by depositing 1 μL droplet of the water-dispersed particles onto 400 mesh Formvar/carbon-supported copper grids (TedPella, Redding, CA, USA). Samples were incubated with the droplets for 1 h followed by gentle removal of the excess solution using filter paper. The TEM grids were air-dried overnight under a fume hood. TEM images were collected using a JEOL-JEM 1400 at 120 kV, utilizing a Gatan camera in conjunction with digital micrograph imaging software.

### 2.2. Scanning Electron Microscopy (SEM)

Samples were first dehydrated using a graded concentration of ethanol. Small droplets of samples in absolute ethanol were then dispersed on appropriate carbon-taped stubs (TedPella, Redding, CA, USA). The samples were then air-dried overnight under a fume hood. To enhance the electron conductivity, samples were coated with gold/palladium (Au/Pd) via sputter coating (Q300T D, Quorum Technologies) and examined using an FEI NanoSEM 450 SEM at 15kV. The compositional characterization of the different particles was performed using energy-dispersive X-ray spectroscopy (EDX), utilizing EDAX^®^ AMETEK^®^ (material analysis division) mounted on the SEM system. The data were analyzed using TEAM™ v4.2.2 software.

### 2.3. Flow Cytometry Analysis and Gating Strategy

Flow cytometry analyses were conducted using standard BD FACSCanto II and Fortessa configurations (BD Biosciences, Franklin Lakes, NJ, USA). The cytometer underwent calibration for FSC resolution using Megamix sizing beads (a fluorescent bead blend with a 2:1:1 ratio of 0.5, 0.9, and 3 µm diameters) and for SSC resolution using Megamix-Plus SSC (a blend of 0.16, 0.20, 0.24, and 0.5µm beads), both acquired from Biocytex, France. For silica-viral-mimicking nanoparticle (VMNP) analysis, voltages were set at FSC = 412 V, SSC = 434 V, and FITC = 654 V. For anti-spike-coated MNP (AS-MNP) characterization, voltages were set at FSC = 209 V, SSC = 357 V, FITC = 286 V, and APC = 300 V.

To eliminate NP doublets and clumps, which could generate a high-fluorescence background and subsequently increase the rate of false positive events, FSC-H vs. FSC-A and SSC-H vs. SSC-A sequential gating was used as a double discrimination strategy. Moreover, to eliminate naked singlet nanoparticles (not involved in viral immuno-capture and thus considered as false negative events), only APC-positive singlets were selected (gated) to determine FITC-positive events. The (%) of double-positive events (APC+/FITC+) was quantified and represented as the MICaFVi value (Figure S1, Supplementary Materials). AS-MNPs incubated with PBS (negative control sample) were used to set the zeroing (background) of the FITC signal.

### 2.4. Western Blotting

Western blotting analysis utilized for detecting the MERS-CoV spike protein involved the addition of 10 µL of 2× Laemmli loading buffer to 10 µL of the supernatant (both pre- and post-conjugation reaction), which was then subjected to SDS-PAGE and transferred onto a polyvinylidene difluoride (PVDF) membrane. Subsequently, the PVDF membrane underwent blocking with 5% bovine serum albumin (BSA) for 1 h at room temperature before being incubated overnight at 4 °C with anti-spike mouse monoclonal antibody (Sinobiological, Beijing, China, cat # 40069-MM23). The membrane was washed thrice (15 min each) with 0.1% PBST and then incubated with the corresponding HRP-conjugated anti-mouse secondary antibody for 1 h at room temperature. Finally, the Clarity Western ECL Substrate (Biorad) was used for membrane incubation, and the ChemiDoc MP imaging system (Biorad) was employed for visualizing the bands.

### 2.5. Synthesis of Spike-Protein-Functionalized Virus-Mimicking Silica Particles (VM-SPs)

First, acid-prepared mesoporous silica microparticles (APMSs) were prepared as previously reported with slight modification [43]. Briefly, CTAB (0.7 g), H_2_O (15 mL), ethanol (5 mL), and concentrated HCl (1.7 mL) were vigorously stirred until the surfactant was dissolved. TEOS (1.4 mL) was then slowly added. After 5 min, NaF (0.5 M in water, 1.85 mL) was added and stirring continued until the mixture turned turbid. The mixture was then quickly transferred to a Teflon bottle and heated at 100 °C for 45 min, filtered, and washed. The resulting silica particles were then acid-functionalized as follows: To 20 mg of APMS suspended in 10 mL of toluene: ethanol (1:1), 40 μL of N-(trimethoxysilylpropyl) ethylenediaminetriacetic acid was added slowly (sequential addition of 10 μL) under nitrogen and the reaction mixture was stirred for 3 h. Acid-functionalized APMSs (APMS-COOH) were then collected via centrifugation, washed with ethanol, acetone, and water several times, and finally redispersed in water. To coat the APMSs with the spike protein, 2 mL of APMS-COOH (5 mg/mL) was added to 200 μL of EDC (14.5 mg) and 200 μL of NHS-sulfo (12.5 mg), and the reaction was rotated for 30 min to activate the carboxylic acids. The recombinant spike protein (25 μg/100 μL) was then added, and after which the reaction was shaken overnight at room temperature. The obtained particles were collected via centrifugation, purified, washed several times with water, and then re-dispersed in water to yield highly stable virus-mimicking silica particles (VM-SPs).

### 2.6. Synthesis of Anti-Spike Antibody-Conjugated Magnetic Nanoparticles (AS-MNPs)

To conjugate the anti-spike antibody to superparamagnetic iron oxide MNPs (size ~500 nm), a covalent amide bond was formed between the amine groups (–NH_2_) of the antibody and the carboxyl groups (–COOH) functionalized on MNPs [44]. Briefly, 2 mL of carboxylic-acid-functionalized MNPs (5 mg/mL) were activated by adding NHS (molar ratio of 1000:1) and EDC (molar ratio of 2000:1) for 20 min in a borate buffer with a pH of 5.5. Following this, the pH was adjusted to 8.0, and 200 µg of anti-spike antibody was added immediately, mixed thoroughly, and incubated for 2 h at room temperature. The resulting anti-spike-conjugated MNPs (AS-MNPs) were magnetically precipitated, washed 3 times with PBS, and then stored at 4 °C. AS-MNPs were very stable and dispersed in their aqueous solutions even after one year of storage.

## 3. The Results and Discussion

### 3.1. MICaFVi Basic Principle

**Figure 1:**
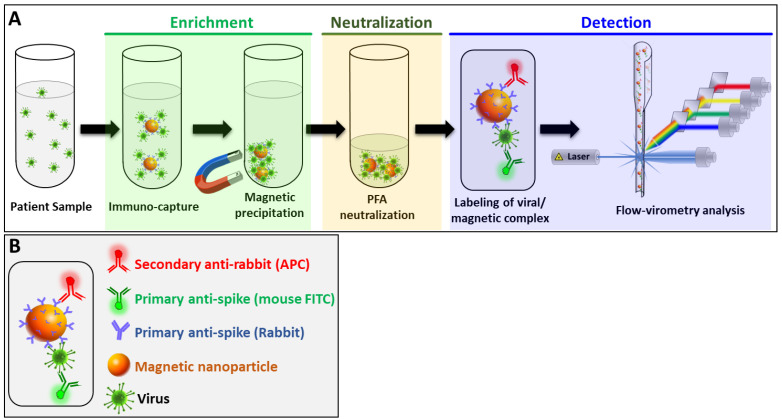
Schematic representation of MICaFVi method. (**A**) In the first step, magnetic-capturing antibody-conjugated MNPs (AS-MNPs) are added to virus-mimicking silica particles (VM-SPs) or spike-protein-expressing pseudoviral particles, incubated, and magnetically captured (enrichment). In the second step, the complex AS-MNP/viral particles are stabilized and neutralized using PFA (neutralization). In the last step, after staining with highly specific fluorescent antibodies, the enumeration of positive AS-MNPs (NPs harboring captured viral particles) is performed via flow cytometry (detection). (**B**) Schematic representation of a positive AS-MNP complexed with a captured virus and stained with two fluorescent antibodies: an APC-labeled antibody used for the staining of AS-MNP and an FITC-labeled anti-spike antibody specific to the staining of the captured virus.

The method of detection developed in this study (MICaFVi) consisted of three main steps: enrichment, neutralization, and detection (Figure 1). The sensitivity of viral diagnostic assays, including RT-qPCR or qPCR, is tightly linked to sample quality; at low viral concentrations, the accuracy of these techniques drops dramatically. To overcome this problem, in the design of our detection method, we included an enrichment step based on viral immuno-capture using iron-oxide MNPs coated with the anti-spike polyclonal antibody (AS-MNPs). Since it is the most immuno-genic and the only surface glycoprotein, the spike protein constitutes the best candidate for coronavirus antigen-based diagnosis [45,46,47,48]. Moreover, the use of polyclonal antibodies in the enrichment step will permit viral immuno-capture with high specificity and sensitivity. Anti-spike antibodies used for immuno-capture were linked to MNPs, permitting magnetic precipitation of the captured viral particles (Figure 1A). The enrichment step concentrates viral particles found in prospective camel/patient samples in a very low volume, and subsequently increases the sensitivity of detection, especially in samples with a low viral titer (early infection). Following the enrichment step, the captured viruses on the surface of AS-MNPs were neutralized by the addition of paraformaldehyde (PFA). This step not only permits the cross-linking of viral particles to AS-MNPs but also the complete neutralization of live viruses, which allows for the safe handling of samples in subsequent analysis steps.

For the detection of captured viral particles, the complex virus/AS-MNPs was stained with a combination of two antibodies: (i) FITC-labeled monoclonal anti-spike antibody, and (ii) a secondary anti-rabbit APC-labeled antibody specific to AS-MNPs (Figure 1B). After the staining step, the AS-MNPs were analyzed via flow cytometry: the detection of double-positive events indicates virus/AS-MNPs complex formation, which demonstrates the presence of a target virus in the analyzed sample. In the case of a viral-free sample, AS-MNPs will only be APC-positive (Figure 1B).

### 3.2. Synthesis and Characterization of VM-SPs

MERS-CoV and SARS-CoV-2 are highly pathogenic viruses that require specific and strict handling precautions (CDC guidelines for MERS-CoV handling). Using these viruses for the design and optimization of a diagnostic assay will be very challenging in terms of safety. Since our detection assay only targets the surface glycoprotein “spike”, and as proof-of-principle, we prepared silica particles coated with this protein (denoted as virus-mimicking silica particles (VM-SPs)) to be used for the optimization of the different steps of our diagnostic technology.

**Figure 2:**
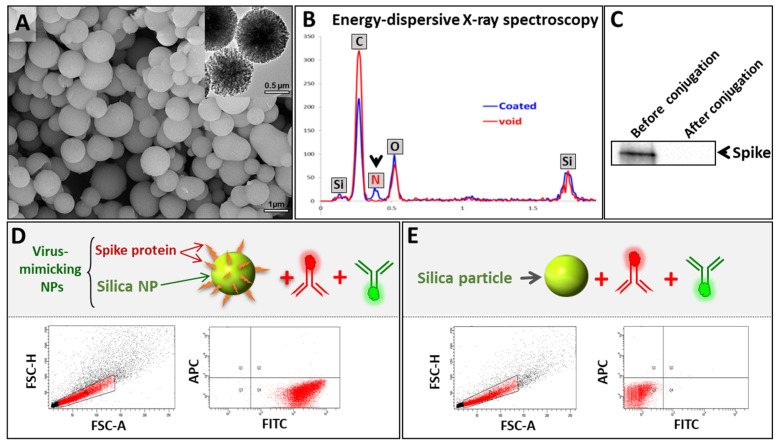
Characterization of virus-mimicking silica particles (VM-SPs). (**A**) SEM and TEM images show uniform average sizes of ~0.5–1 µm. (**B**) EDX spectroscopy showing the chemical composition of naked and spike-protein-coated SPs. SPs showed an extra peak corresponding to the nitrogen element which indicates the successful attachment of the spike protein on their surface. (**C**) Western blotting analysis was used to quantify the spike protein in the reaction supernatant before and after conjugation. After conjugation, the spike protein was not detected in the supernatant, indicating complete protein conjugation. (**D**) Flow cytometry analysis of VM-SPs using an FITC-labeled anti-spike protein clearly showed that almost all SPs were FITC-positive (coated with spike protein). A non-specific APC-labeled antibody was also used as a negative control. (**E**) To eliminate any possible non-specific binding of the FITC-anti-spike antibody to SPs directly, we used this antibody to stain the naked SPs. The results showed that all events were negative.

To synthesize VM-SPs, acid-functionalized mesoporous silica particles (APMS-COOH) were first prepared followed by amide coupling with the free amine groups of the MERS-CoV spike protein, leading to spike-protein-conjugated SPs. The as-synthesized viral particles were characterized using electron microscopy (SEM and TEM) to examine their morphology and size. As expected, VM-SPs showed ~0.5–1 µm uniform sizes (Figure 2A and Figure S2). We confirmed the conjugation of the spike protein to the silica particles via several methods. The results of the comparative analysis of VM-SPs before and after coating via energy-dispersive X-ray (EDX) spectroscopy showed the presence of an extra element in the composition of the coated particles corresponding to nitrogen. The presence of this element is indirect proof of the presence of the spike protein on SPs (Figure 2B). Moreover, the detection of the spike protein via Western blotting analysis showed that the spike disappeared completely from the supernatant after the coating reaction, indicating quantitative protein conjugation to acid-functionalized silica microparticles (Figure 2C). To provide direct evidence of spike protein attachment, SPs were stained with an FITC-labeled anti-spike monoclonal antibody and then analyzed using flow cytometry. The results showed that all of the SPs were coated with the spike protein (FITC-positive) (Figure 2D). To exclude the hypothesis that the FITC-antibody stains the core of SPs via non-specific binding, we used naked non-functionalized SPs that were stained similarly, and no FITC-positive events were detected. This confirms that the FITC-labeled anti-spike antibody was attaching to SPs via the spike protein (Figure 2E). It is noteworthy that the anti-rabbit APC-labeled secondary antibody that was used in the subsequent steps of MICaFVi was added during these tests to exclude any non-specific interaction between either the FITC-labeled antibody or the silica particles (Figure 2D,E).

### 3.3. Synthesis and Characterization of AS-MNPs

**Figure 3:**
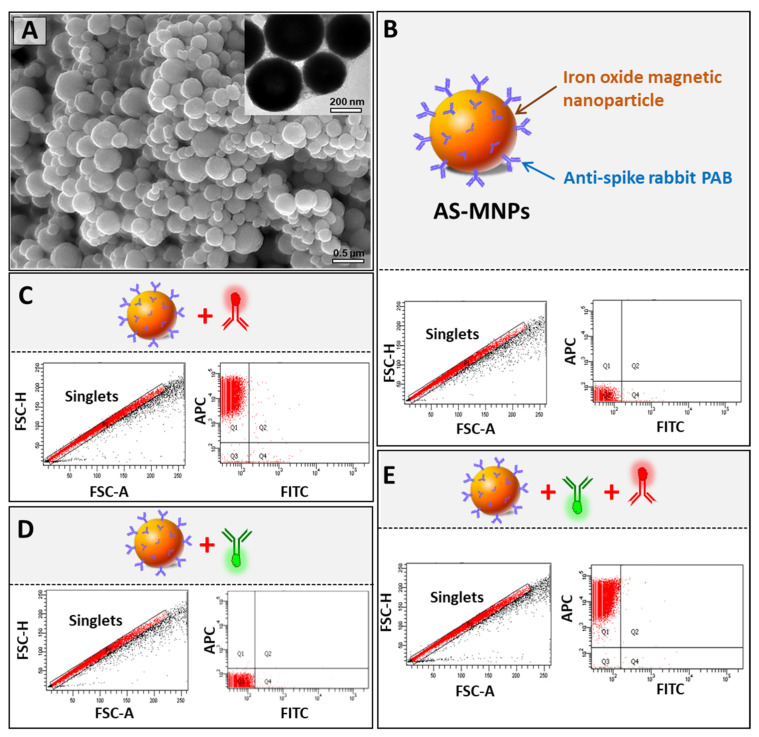
Characterization of anti-spike Ab-coated MNPs (AS-MNPs). (**A**) SEM and TEM images of AS-MNPs showing 500 nm sized MNPs. (**B**) Schematic representation and behavior when analyzed via flow cytometry. Non-stained AS-MNPs were used to set up the zeroing of fluorescence signals (FITC and APC) during flow cytometry analysis. Only singlets were gated for analysis. (**C**) Analysis of the coating efficiency of AS-MNPs via the anti-spike rabbit pAb (blue) using an APC-labeled anti-rabbit secondary Ab (red). (**D**) Analysis of the interaction between the FITC-labeled anti-spike Ab (green) with AS-MB. (**E**) Analysis of the interaction between APC-labeled anti-rabbit secondary Ab (red) and the FITC-labeled anti-spike Ab (green).

In our diagnostic assay, the immuno-capture of viral particles, during the enrichment step, was performed using superparamagnetic MNPs coated with rabbit polyclonal anti-spike antibody. Such superparamagnetic NPs respond rapidly to an applied magnetic field but exhibit negligible residual magnetism away from the magnetic field, making them particularly attractive for biodetection applications [32]. To assess their quality, AS-MNPs were characterized using SEM, TEM, and flow cytometry. SEM and TEM imaging clearly showed that the majority of AS-MNPs had a regular spherical shape with an average diameter of 500 nm (Figure 3A and Figure S2). The efficiency of AS-MNPs in the immuno-capturing of the virus-mimicking particles is tightly dependent on both the quantity of antibodies on their surface and the quantity of spike proteins on the surface of VM-SPs. To ensure that they were efficiently coated, AS-MNPs were stained with an APC-labeled secondary anti-rabbit antibody and analyzed using flow cytometry. Non-stained MNPs were used as a control for gate setting (Figure 3B). Flow cytometry analysis showed that 100% of the stained AS-MNPs were coated with anti-spike antibodies (all events were APC-positive) (Figure 3C). Since different antibodies were used in MICaFVi (FITC-labeled monoclonal anti-spike antibody, APC-labeled secondary anti-rabbit antibody, and anti-spike rabbit polyclonal antibody) (Figure 1B), we performed different tests to ensure that no non-specific interactions existed between these elements, as such interactions would have increased false positive events during diagnosis and subsequently affected the assay specificity and accuracy. To exclude any possible non-specific interaction between AS-MNPs and the FITC-labeled monoclonal anti-spike antibody, used for the detection of immuno-captured viral particles, these two components were incubated together and AS-MNPs were analyzed using flow cytometry. The results showed that almost all AS-MNPs were FITC-negative, which excludes any non-specific interaction between these two components (Figure 3D). To exclude the hypothesis that FITC-labeled monoclonal anti-spike antibody could bind to AS-MNPs through the APC-labeled secondary anti-rabbit antibody, these three elements were incubated together and then AS-MNPs were analyzed using flow cytometry. The results showed again the absence of FITC-positive events, which indicates that the FITC-labeled monoclonal anti-spike antibody interacted neither with AS-MNPs nor with the APC-labeled secondary anti-rabbit antibody (Figure 3E).

### 3.4. Optimization of MICaFVi

**Figure 4:**
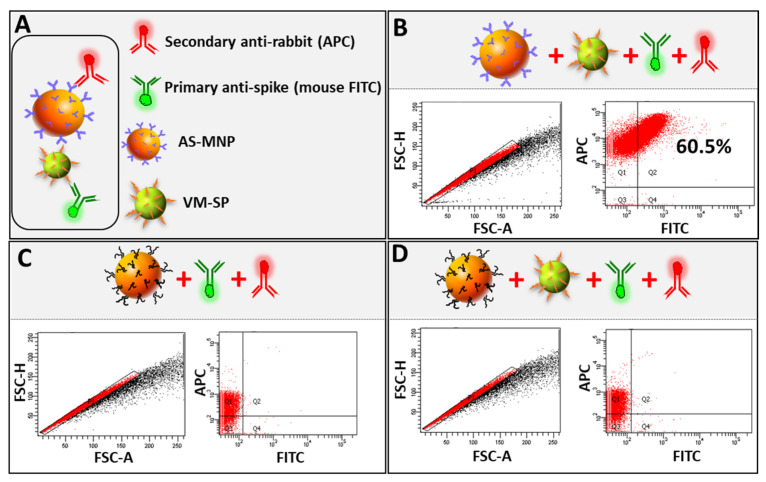
MICaFVi detection of VM-SPs. (**A**) Viral capture using AS-MNPs is mediated via a specific interaction between anti-spike antibodies and the spike protein on VM-SPs. Legend and representation of fluorescent antibodies used to stain captured VM-SPs via AS-MNPs. (**B**) The efficiency of AS-MNPs in capturing the VM-SPs. (**C**) Heat-denaturation of AS-MNPs in Laemmli does not strip anti-spike Abs (AS-MNPs are still APC-positive after denaturation) but only denatures them. (**D**) VM-SP immuno-capture is specifically mediated by the interaction between anti-spike antibodies (on the surface of AS-MNPs) and spike protein (on the surface of VM-SPs). Denatured AS-MNPs failed to capture VM-SPs.

Having characterized all of the components of MICaFVi, we optimized the protocol as follows. VM-SPs and AS-MNPs were co-incubated overnight to allow for immuno-capture (Figure 4A). The AS-MNPs were then magnetically captured via a multi-well magnetic separator and then incubated in 4% PFA for 30 min. This step was crucial for the stabilization of the AS-MNPs/VM-SPs complex during the subsequent analysis steps. Moreover, incubation with PFA allows for the neutralization of wt MERS-CoV/SARS-CoV-2, for when camel/human samples will be analyzed in subsequent studies. Following the neutralization/fixation step, the magnetically captured AS-MNPs were washed 3 times in PBS with 0.3% (*v*/*v*) tween to remove any non-specific bound interactions. The precipitated AS-MNPs were then stained with FITC-labeled mouse monoclonal anti-MERS-CoV spike antibody and APC-labeled secondary anti-rabbit antibody at a dilution of 2/1000 in PBS containing 1% tween and 50% FBS for 1h. Stained AS-MNPs were magnetically precipitated, then washed 3 times with PBS containing 0.3% (*v*/*v*) tween, and analyzed using flow cytometry (Figure 4B). We followed a very strict gating strategy to eliminate doublets and clumps, as well as false positive and false negative events (see Section 2 and Figure S1). AS-MNPs incubated with PBS (negative control sample) were used to set the zeroing (background) of the fluorescent signal (FITC and APC). The number of double-positive events (representing the complex AS-MNPs/VM-SPs) was quantified and represented as a % of MICaFVi. The results showed that ~ 60% of AS-MNPs were FITC-positive (Figure 4B), which indicates that they were cross-linked (captured) with the viral particles. To prove that the formation of the AS-MNPs/VMNPs complex was the result of a specific interaction between the spike protein (on the surface of VM-SPs) and its antibody (on the surface of AS-MNPs), and to exclude any possibility of non-specific cross-linking, AS-MNPs were boiled in Laemmli buffer to completely denature the anti-spike antibodies on their surface. These boiled AS-MNPs were used to immuno-capture VM-SPs. Flow cytometry analysis showed that boiled AS-MNPs were still APC-positive to a lesser extent, due to the binding of the APC-labeled secondary antibody to the denatured primary antibody (Figure 4C). No FITC-positive events were recorded, indicating the failure of AS-MNPs/VM-SPs complex formation (Figure 4D). This confirms that the interaction between the spike protein and its antibody on the surface of AS-MNPs is crucial for immune capture (complex formation).

**Figure 5:**
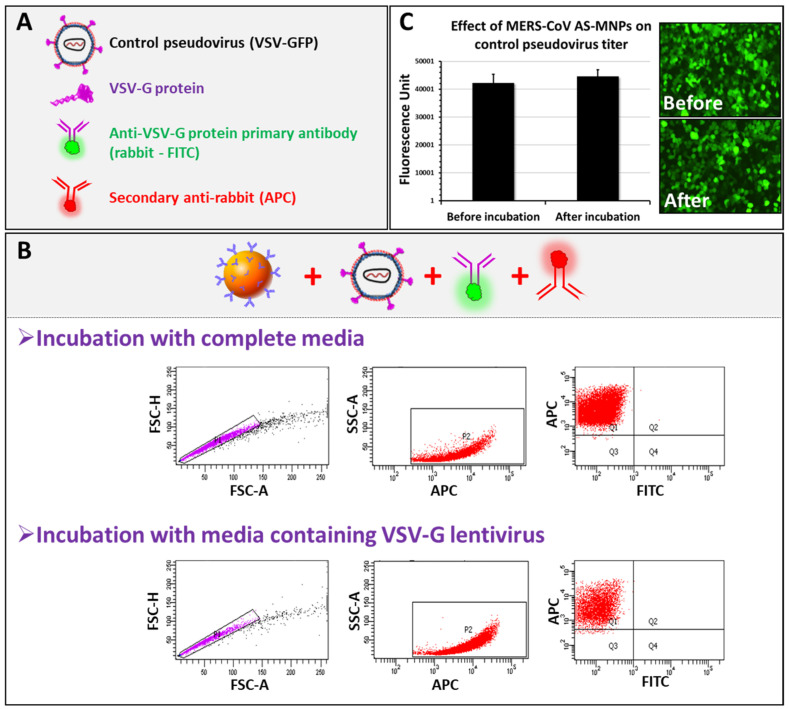
MICaFVi specificity. (**A**) Legend. (**B**) MERS-CoV AS-MNPs failed in capturing VSV-Gpp. (**C**) VSV-Gpp viral titer (fluorescence unit) remained unchanged before and after incubation with MERS-CoV AS-MNPs.

To evaluate the specificity of the assay, the VSV-Gpp-pseudotyped lentivirus expressing the VSV-G protein on its surface was used. We performed MICaFVi using an FITC-labeled primary anti-VSV-G protein antibody in combination with the anti-Rabbit APC-labeled secondary antibody (Figure 5A). Complete cell culture medium (virus-free) was used as a negative control. Flow cytometry analysis of AS-MNPs, incubated with VSV-Gpp, showed that all events were APC-positive and FITC-negative (Figure 5B). These results indicate that AS-MNPs failed to capture VSV-Gpp, which confirms the specificity of these NPs to MERS-CoV spike protein detection.

To further show that no VSV-Gpps were depleted by AS-MNPs, viral solutions, before and after incubation with AS-MNPs, were used to infect Huh7 cells. VSV-Gpp carries a GFP reporter gene used to quantify the viral titer. The infection assay results showed no difference in fluorescence intensity before and after incubation with AS-MNPs (Figure 5C), which indicates that the viral titer remained unchanged after incubation with AS-MNPs. In other words, no VSV-G particles were recognized and captured by these nanoparticles. Taken together, our results strongly illustrate the high specificity of AS-MNPs for the capture of the intended target (spike protein).

### 3.5. MICaFVi Detection of MERS-CoV Pseudoviral Particles (MERSpp)

**Figure 6:**
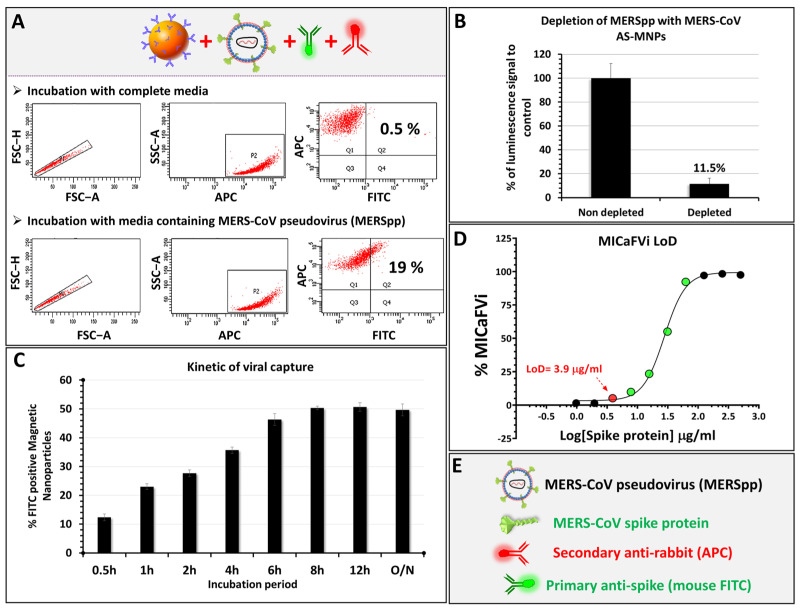
MICaFVi detection of pseudovirus. (**A**) Detection of MERS-CoV pseudoviral particles using MICaFVi. Complete cell culture medium was used as a negative control. (**B**) Evaluation of AS-MNPs’ immuno-capture potential via their viral depleting effect. (**C**) Time-dependent sensitivity of MICaFVi. (**D**) MICaFVi limit of detection (LoD) was determined using different concentrations of MERS-CoV-free spike protein. (**E**) Legend.

We showed that MICaFVi detects coronavirus spike proteins on the surface of virus-mimicking particles efficiently and specifically. To demonstrate that our assay is also applicable to the detection of viral particles, we tested its ability in detecting a pseudovirus expressing the MERS-CoV spike protein on its surface (i.e., MERSpp) (Figure 6). The results of the flow cytometry analysis of AS-MNPs incubated with a solution of MERSpp showed that 19% of events were APC/FITC double-positive, confirming that our method successfully detected MERSpp present in the used sample. Virus-free cell culture medium was used as a negative control (Figure 6A). To further confirm the immuno-capture of the pseudoviral particles, we performed a luciferase assay using an MERSpp-carrying luciferase reporter gene. Huh7 cells were infected with pseudovirus solution before or after depletion with AS-MNPs. The results of luminescence quantification, after viral infection, showed that incubation with AS-MNPs reduced the luminescence to 11.5% compared to the control (Figure 6B). This indicates that the majority (88.5%) of MERSpp in the depleted solution was immuno-captured.

The turnaround time of diagnostic tests is extremely important for patient management, particularly during outbreaks and pandemics. In such a context, high-throughput systems along with rapid diagnostic testing are essential. Thus, to define the minimum incubation time needed for optimal sensitivity, we incubated AS-MNPs with a solution of MERSpp, and MICaFVi was then performed at different time points of incubation. The results showed that MICaFVi was able to detect viral particles (positive signal) with an incubation period as short as half an hour (% double-positive events ~14%). The highest double-positive events (50% MICaFVi) were reached after 6–8 h of incubation (Figure 6C). However, even with a reduced incubation time, MICaFVi can still detect viral particles, albeit with lower sensitivity. This means that for samples with a high viral titer, half an hour will be sufficient to detect the virus in the sample; however, samples with a lower viral titer will need an extended incubation time to reach the optimal sensitivity.

The sensitivity of MICaFVi was experimentally determined using the free recombinant spike protein. MICaFVi was carried out to capture the free spike protein from different known dilutions. The results showed that the minimum concentration detected was 3.9 µg/mL of spike protein (MW ~200 kDa), resulting in a limit of detection (LOD) equal to 20 pmol/mL (Figure 6D). It has to be noted that several factors can contribute to the specificity and sensitivity of MICaFVi detection. These factors encompass the quality and specificity of the capture antibodies utilized, the quality and stability of the sample or pseudoviral particles being analyzed, the efficiency of the immuno-capture process, and, most importantly, the physiochemical features of the functional MNPs that capture the virus (i.e., size, stability, appropriate functionality, and magnetic responsiveness). The optimization of all of these factors is necessary to attain the precise, accurate, and dependable detection of the target virus. Finally, MICaFVi constitutes a very flexible technique that could be easily adapted for diverse pathogen detection (viruses, bacteria, proteins, and even cells) (Figure S3) either separately or simultaneously (Multiplex MICaFVi).

## 4. Conclusions

In conclusion, we developed a novel magnetic immuno-capture approach, MICaFVi, for the detection of viral-mimicking and pseudoviral particles as surrogates for MERS-CoV and SARS-CoV-2. MICaFVi is based on capturing the spike proteins exposed on coronaviruses using anti-spike antibody-conjugated MNPs (AS-MNPs), followed by the use of a flow cytometer to detect viral particles (i.e., VM-SPs or pseudoviral particles), enabling efficient, fast, accurate, sensitive, and specific detection. Our method combines the high specificity and efficiency of the capturing functionalized MNPs with the high sensitivity and accuracy of flow cytometry. MICaFVi detected successfully mimic-virus silica particles as well as MERS-CoV pseudoviral particles (MERS-pp) with high efficiencies of ~60% and 90%, respectively, and LOD = 20 pmol/mL. The proposed approach has great potential for designing an alternative approach for the accurate and sensitive diagnosis of SARS-CoV-2 and other viral infections. Moreover, MICaFVi can be adaptable for diverse pathogen detection either separately or simultaneously (Multiplex MICaFVi), and could be potentially used to detect other viruses, bacteria, proteins, and even cells.

## Data Availability

The original data presented in the study are included in the article/Supplementary Material; any further data inquiries can be directed to the corresponding author.

## Supplementary Materials

The following supporting information can be downloaded at https://www.mdpi.com/article/10.3390/bios13050553/s1, Figure S1: Gating strategy: The first steps consist of NP visualization based on their sorting according to their forward and side scatter properties. To eliminate NP doublets and clumps, that could generate a high fluorescence background and subsequently increases the rate of false positive events, FSC-H vs FSC-A and SSC-H vs SSC-A sequential gating was used as a double discrimination strategy; Figure S2: SEM and TEM images of virus-mimic silica particles (VM-SPs) and anti-spike protein conjugated (AS-MNPs) showing their sizes and morphology; Figure S3: Adaptability of MICaFVi technique that could be easily applied for diverse pathogen detection (viruses, bacteria, proteins, and even cells).
