# Supplementary material for: MICaFVi: A Novel Magnetic Immuno-Capture Flow Virometry Nano-Based Diagnostic Tool for Detection of Coronaviruses
Source: Biosensors (Basel). 2023 May 18;13(5):553. doi: 10.3390/bios13050553 (PMC10216117; doi:10.3390/bios13050553)
Supplement: Supplementary file 1 [file biosensors-13-00553-s001.zip › biosensors-2340819-supplementary.pdf]

biosensors

Supplementary Materials

MICaFVi: A Novel Magnetic Immuno-Capture Flow Virometry Nano-Based Diagnostic Tool for Detection of Coronaviruses

Nosaibah Samman, Kheireddine El-Boubbou, Khawlah Al-Muhalhil, Rizwan Ali, Ahmad Alaskar, Naif Khalaf Alharbi and Atef Nehdi

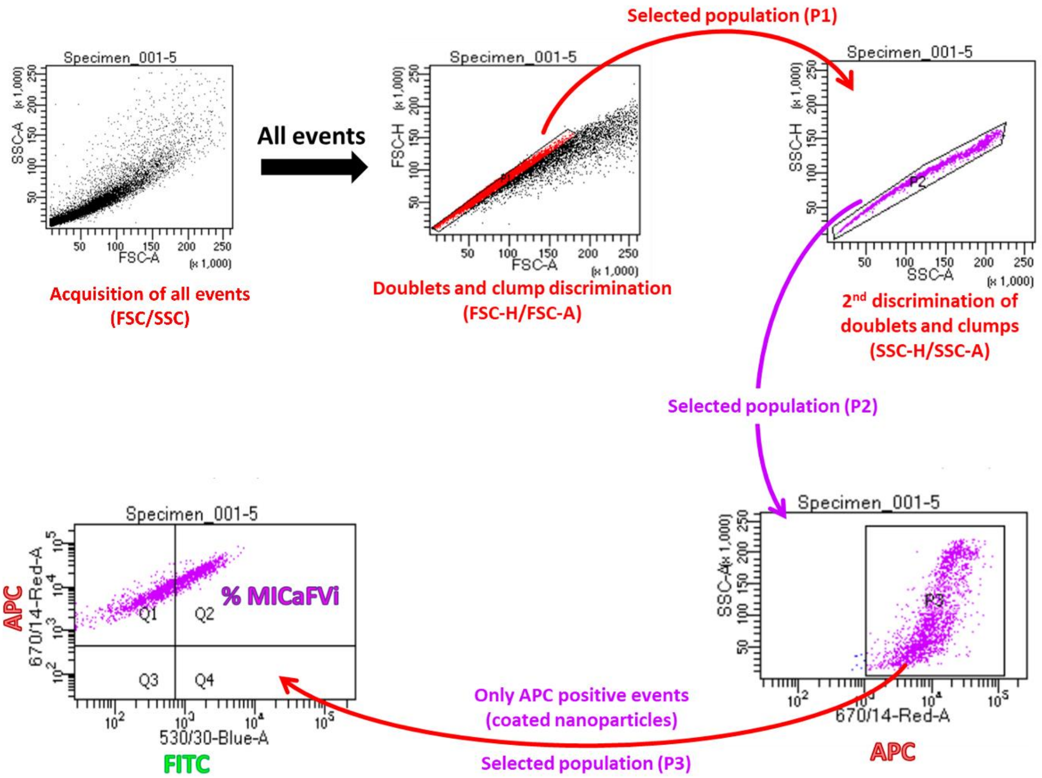

Figure S1. Gating strategy: The first steps consists of NP visualization based on their sorting according to their forward and side scatter properties. To eliminate NP doublets and clumps, that could generate a high fluorescence background and subsequently increases the rate of false positive events, FSC-H vs FSC-A and SSC-H vs SSC-A sequential gating was used as a double discrimination strategy. The population of single nanoparticle (P2), was than subjected to a third selection based on APC signal, all APC-negative NPs (naked NPs) were excluded because they are not able to capture viral particles and thus, if not eliminated, will increase the rate of false negative events. Only APC-positive events (P3) were gated and used to determine the (%) of FITC positive events, double positive events (APC+/FITC+) constitutes MICaFVi value.

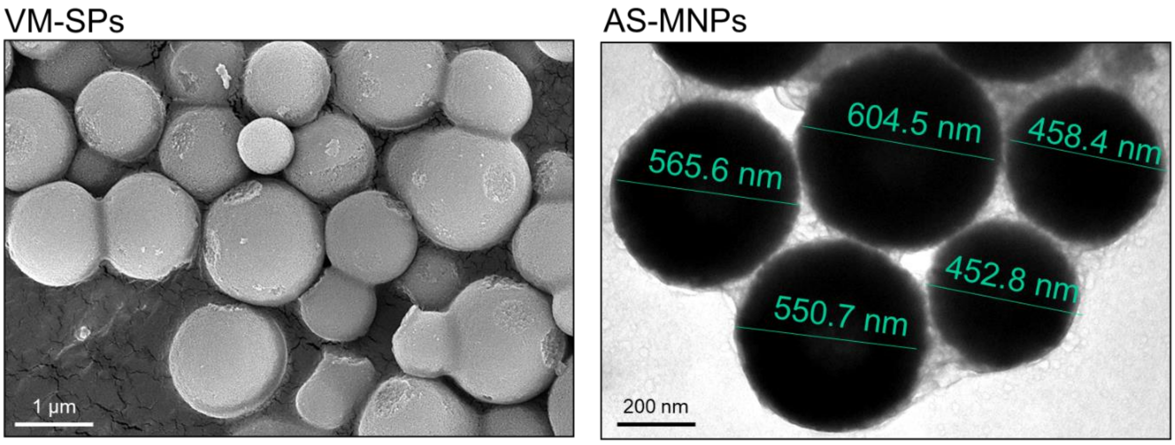

Figure S2. SEM and TEM images of virus-mimic silica particles (VM-SPs) and anti-spike protein conjugated (AS-MNPs) showing their sizes and morphology.

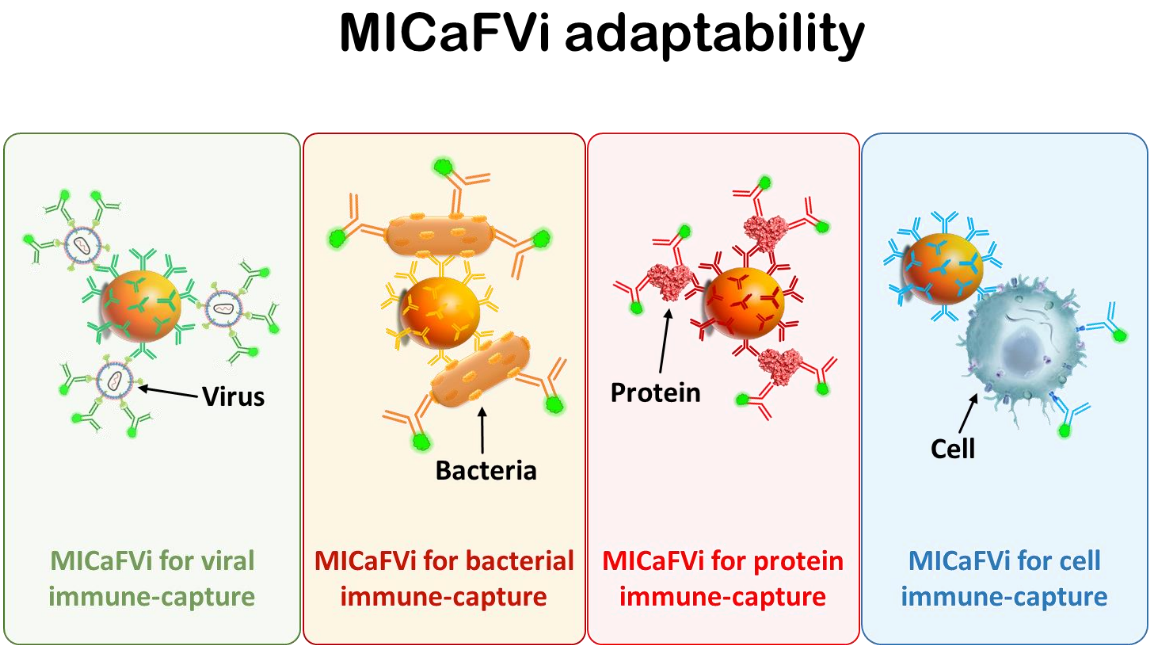

Figure S3. Adaptability of MICaFVi technique that could be easilly applied for diverse pathogen detection (viruses, bacteria, proteins, and even cells).
